# Dispersant Enhances Hydrocarbon Degradation and Alters the Structure of Metabolically Active Microbial Communities in Shallow Seawater From the Northeastern Gulf of Mexico

**DOI:** 10.3389/fmicb.2019.02387

**Published:** 2019-10-18

**Authors:** Xiaoxu Sun, Lena Chu, Elisa Mercando, Isabel Romero, David Hollander, Joel E. Kostka

**Affiliations:** ^1^School of Earth and Atmospheric Sciences, Georgia Institute of Technology, Atlanta, GA, United States; ^2^Guangdong Key Laboratory of Agricultural Environment Pollution Integrated Control, Guangdong Institute of Eco-Environmental Science and Technology, Guangzhou, China; ^3^School of Biological Sciences, Georgia Institute of Technology, Atlanta, GA, United States; ^4^College of Marine Science, University of South Florida, St. Petersburg, St. Petersburg, FL, United States

**Keywords:** hydrocarbon degradation, Deepwater Horizon, dispersant, microbial community, hydrocarbon analysis

## Abstract

Dispersant application is a primary emergency oil spill response strategy and yet the efficacy and unintended consequences of this approach in marine ecosystems remain controversial. To address these uncertainties, *ex situ* incubations were conducted to quantify the impact of dispersant on petroleum hydrocarbon (PHC) biodegradation rates and microbial community structure at as close as realistically possible to approximated *in situ* conditions [2 ppm v/v oil with or without dispersant, at a dispersant to oil ratio (DOR) of 1:15] in surface seawater. Biodegradation rates were not substantially affected by dispersant application at low mixing conditions, while under completely dispersed conditions, biodegradation was substantially enhanced, decreasing the overall half-life of total PHC compounds from 15.4 to 8.8 days. While microbial respiration and growth were not substantially altered by dispersant treatment, RNA analysis revealed that dispersant application resulted in pronounced changes to the composition of metabolically active microbial communities, and the abundance of nitrogen-fixing prokaryotes, as determined by qPCR of nitrogenase (*nifH*) genes, showed a large increase. While the *Gammaproteobacteria* were enriched in all treatments, the *Betaproteobacteria* and different families of *Alphaproteobacteria* predominated in the oil and dispersant treatment, respectively. Results show that mixing conditions regulate the efficacy of dispersant application in an oil slick, and the quantitative increase in the nitrogen-fixing microbial community indicates a selection pressure for nitrogen fixation in response to a readily biodegradable, nitrogen-poor substrate.

## Introduction

The Deepwater Horizon (DWH) oil spill represents the largest accidental marine oil spill in history (Farrington, 2013; Sammarco et al., 2013). A total of 3.19 million barrels of crude oil along with an unprecedented 1.84 million gallons of dispersant, COREXIT 9500A and COREXIT 9527, were released into the Gulf of Mexico (GOM) (Barbier, 2015). Nearly 60% of the dispersant was applied at the sea surface. Dispersants are complex mixtures of chemical surfactants in a hydrocarbon solvent – the surfactants lower the interfacial tension between oil and water, breaking the oil into small droplets, with an increased surface-to-volume ratio and availability for biodegradation (National Research Council, 2005; Prince, 2015).

Although biodegradation has been studied for decades in seawater, there is as yet no consensus on the rates and controls of the process (Prince et al., 2017). The rate of biodegradation is limited by a range of environmental parameters including oxygen (Leahy and Colwell, 1990), temperature (ZoBell, 1969), nutrient availability (Atlas and Bartha, 1972), pressure (Schwarz et al., 1975; Prince et al., 2016b), and microbial community composition (Joye et al., 2016). Moreover, recent studies reveal that the concentration and form of oil (slick or water accommodated fractions) used in experiments is a major confounding factor in our understanding of biodegradation (Kleindienst et al., 2015b; Prince et al., 2017). The biodegradation rate of dispersed oil slows as the concentration of oil is increased and autochthonous nutrients become limiting at oil concentrations above 250 ppm (Prince et al., 2017). Although dispersant application is widely used as an emergency response strategy, the efficacy of this approach remains under debate (Kleindienst et al., 2015a; Prince et al., 2017). Previous studies indicate that dispersant can enhance (Venosa and Holder, 2007), have little effect (Foght and Westlake, 1982), or diminish (Lindstrom and Braddock, 2002) the rate and extent of petroleum hydrocarbons (PHCs) degradation in seawater. The range in the enhancement of biodegradation by dispersant can be explained in part by differences in the design and approach of previous experiments (Prince and Butler, 2014; Kleindienst et al., 2015a). Experiments must be conducted with concentrations of oil/dispersant resembling those expected during emergency response to an oil spill (Lee et al., 2013). During the DWH discharge, oil concentrations varied from below detection to over 10,000 ppm in surface seawater in the GOM, and the concentration was rapidly diluted with distance from the wellhead (Sammarco et al., 2013). In a survey conducted by NOAA immediately after the DWH oil spill, the majority of observations in the water column were at or below the low ppm level (U. S. National Oceanic and Atmospheric Administration [NOAA], 2010; Wade et al., 2016). At these low ppm concentrations of oil, bioavailable nutrients and oxygen are unlikely to limit microbial activity (Edwards et al., 2011). In contrast, the majority of previous experiments quantified biodegradation in seawater incubations using oil concentrations which are orders of magnitude higher than those expected during active response [i.e., 125 – 2500 ppm (Zahed et al., 2010), 867 ppm (Campo et al., 2013), 83 and 833 ppm (Venosa and Holder, 2007), and 1400 ppm (Lindstrom and Braddock, 2002)]. Using such high oil concentrations in a closed system may cause depletion of nutrients and artifacts which hinder biodegradation (Lee et al., 2013). Further, the dispersion process depends on oceanic mixing conditions, and lab incubations should reflect the mixing conditions in the environment (Venosa et al., 2002; Prince and Butler, 2014). Another uncertainty of dispersant application is the potential impact on indigenous microbial communities (Kleindienst et al., 2015a). Previous studies come to equivocal conclusions by suggesting that dispersants may impede or stimulate the activity of microbial populations (Lindstrom and Braddock, 2002; Chakraborty et al., 2012; Kleindienst et al., 2015b).

The objectives of this study were to investigate the biodegradation of dispersed oil in relation to mixing condition as well as the impacts of dispersant on the dynamics of metabolically active microbial communities in coastal seawater. Surface seawater was amended with artificially weathered Macondo surrogate oil and Corexit 9500A dispersant under as close to *in situ* conditions as possible. Petroleum hydrocarbon degradation and microbial communities were characterized using a close coupling of analytical chemistry and next generation sequencing techniques. Sequencing analysis of extracted RNA was used as a proxy for the metabolically active microbial communities. The results point to the importance of mixing conditions and alteration of metabolically active microbial communities following dispersant application. Our results under environmentally relevant conditions can be used to improve models that predict the fate of dispersed oil during future emergency response efforts.

## Materials and Methods

### Sample Collection and Experimental Setup

Surface seawater (top 1 m) was collected from Pensacola beach, FL (30°19′ N, 87°10′ W) with carboys for low mixing and high mixing experiments in June and August 2016, respectively. The sampling site was described in a previous study (Kostka et al., 2011). Seawater was immediately transported to the lab and aerated overnight. Microcosm experiments were conducted as previously described by Prince and Butler with the modifications described below (Prince and Butler, 2014). Two treatments were tested: seawater amended with weathered oil (oil treatment) or seawater amended with weathered oil and dispersant (oil + dispersant treatment) (COREXIT EC9500A; Nalco Environmental Solutions LLC, TX, United States). Surrogate MC252 oil was weathered by evaporation in the lab according to previously published methods (Pelz et al., 2011; Prince and Butler, 2014). Similar to previous work, we gravimetrically measured a 20% weight loss after the weathering process (Prince and Butler, 2014). The chemical composition of the surrogate MC252 crude oil was not confirmed prior to weathering. However, the chemical composition of the surrogate oil has been well characterized in previous work (Pelz et al., 2011) and shown to be highly similar to the MC252 oil, which both contain roughly 20% volatile organic carbon. Analysis of PHCs at the beginning of the incubations confirmed that most volatile PHCs had been removed, and thus, it was concluded that volatile compounds constituted a minor fraction of the weathered oil used as corroborated by previous work (Pelz et al., 2011). Nonetheless, we acknowledge that hydrocarbons in the low range of molecular weight (e.g., C12–C19 alkanes, two and three ring-aromatics), present at the beginning of our incubations, could have been affected by evaporation. Two experiments were conducted under identical conditions, with the exception that the mixing condition was varied. For all enrichments, 1.5 L of seawater was incubated in 2 L glass bottles. In oil treatments under both mixing regimes, 3 μl of oil was added to a floating boom to create a surface oil slick (Supplementary Figure S1; Prince and Butler, 2014). For the low-mixing oil + dispersant treatment, 3 μl of weathered oil was added to a floating boom, followed by 0.2 μl of dispersant. For the high-mixing oil + dispersant treatment, 3.2 μl of premixed oil/dispersant mixture was added to the bottles without booms. The mixture was prepared with a dispersant to oil ratio (DOR) of 1:15, similar to that recommended for emergency oil spill response (Joint Analysis Group [JAG], 2010; Prince and Butler, 2014; Kleindienst et al., 2015a). For each mixing condition, a total of 48 2L bottles were incubated for both chemical (24 bottles) and biological (24 bottles) analysis. Our previous incubations of surface GOM seawater revealed little to no microbial activity detected as respiration to carbon dioxide when no oil was added.

Bottles were incubated unsealed in an incubator at 100 RPM and 25°C in the dark. Autoclaved Nanopure water was routinely added to compensate for evaporation. Based on previous research, the evaporation of weathered oil is expected to be minimal (Prince and Butler, 2014). True duplicate samples for each treatment were sacrificed for both nucleic acid and oil extraction at 0, 7, 15, 22, 30, and 40 days. Additional duplicate bottles were prepared for each treatment and sealed with rubber stoppers for quantification of respiration. Oxygen concentrations were measured by a Presens MicroX 4 optode system with PSt7 non-invasive sensor spots (Presens, Germany) adhered to the inside of the glass bottle. Carbon dioxide concentrations were measured by injecting 100 μl of headspace into a GC-FID equipped with a methanizer (Shimadzu, Japan). Nutrient concentrations were monitored throughout the incubation period. Nitrite/nitrate, ammonia and soluble phosphate measurements were determined using established methods (Murphy and Riley, 1962; Strickland and Parsons, 1972; García-Robledo et al., 2014).

### Extraction and Analysis of Hydrocarbons

At each time point, bottles were frozen at −20°C until extraction. For the low-mixing treatments, enrichments were extracted and analyzed according to Prince and Butler (2014). Briefly, the entire volume of each bottle was extracted with chloroform and the solvent was concentrated under a nitrogen purge before analysis. The high-mixing scenario samples were extracted as follows. A 10 μl aliquot of mixed standard was added to each bottle to correct for extraction efficiency. A 200 ml volume of 100% ACS grade dichloromethane (DCM) (BDH Chemicals, United Kingdom) was added to each bottle and gently shaken for 30 s. The DCM layer was then carefully transferred to a separatory funnel and collected in a flask from the bottom. DCM treatment was repeated three times to recover all the residual oil from the bottle. The retrieved DCM layer was then reduced in volume to 5 ml in a Turbovap (Biotage, Uppsala, Sweden) followed by addition of 20 ml of 100% ACS grade hexane (Fisher Chemical, NJ, United States). The volume of the extract was further reduced to 1 ml and stored in brown glass HPLC vials at 4°C until analysis. Extracted oil samples from low-mixing enrichments were analyzed by GC-MS as previously described (Prince and Butler, 2014). The high-mixing samples were analyzed based on previous literature with optimization (Romero et al., 2015; Sørensen et al., 2016). Briefly, samples were analyzed on a GC/MS/MS with a quadrupole mass spectrometer (Agilent, CA, United States), following EPA methods (Method 8270D). DWH crude oil (NIST 2779) was used as a reference standard. The fact that oil measurements were conducted with different instruments at separate institutions was unfortunate and caused by the limited availability of instrument time. Thus, direct comparisons were avoided between the two mixing conditions.

Since PHCs were extracted with different protocols and analyzed on different GC-MS instruments for the two experiments, results are reported as relative values to T0, with respect to 17α(H),21β(H)-hopane as a conserved internal standard (Prince et al., 1994). Half-lives of each class of PHCs were calculated using a first order kinetic equation (Edwards et al., 2011; Zahed et al., 2011; Prince et al., 2013).

### Nucleic Acid Extraction and Microbial Community Characterization

Only the high mixing scenario samples were extracted and analyzed for microbial community characterization. All 1.5 L of seawater from each bottle was filtered through 0.2 μm Mobio Powerwater Filter (MoBio Laboratories, CA, United States) and stored at −80°C before extraction. Each half of the filter was extracted for DNA and RNA using the Mobio Powerwater DNA isolation kit (MoBio Laboratories, CA, United States) and Mobio Powerwater RNA isolation kit (MoBio Laboratories, CA, United States), respectively, following the manufacturer’s protocols. RNA was reverse-transcribed to cDNA using the qScript XLT cDNA supermix kit (Quantabio, MA, United States). Extracted DNA or cDNA was quantified with the Qubit HS assay kit (Invitrogen, Carlsbad, CA, United States) and 10 ng per reaction was used to generate SSU rRNA amplicons. Prokaryotic community composition was determined by applying a high-throughput sequencing-based protocol that targets PCR-generated amplicons from the V4 variable regions of the SSU rRNA gene using the primer set CS1_515F (5′-CACTGACGACATGGTTCTACA_GTGCCAGCMGCCGC GGTAA) and CS2_806R (5′-TACGGTAGCAGAGACTT GGTCT_GGACTACHVGGGTWTCTAAT) (Caporaso et al., 2010; Moonsamy et al., 2013). The resulting SSU rRNA gene amplicons were barcoded with unique 10-base barcodes (Fluidigm Corporation, CA, United States), pooled into equal DNA aliquots, and sequenced on an Illumina MiSeq2000 platform at the DNA services facility of the University of Chicago according to established methods (Bybee et al., 2011; Green et al., 2015; Herbold et al., 2015). The generated sequence libraries are available at NCBI under BioProject: PRJNA434233.

Sequence libraries were processed using multiple bioinformatics tools. Paired-end reads were merged with PEAR. The merged sequences were then demultiplexed and trimmed with vsearch and mothur, respectively. Chimeras were detected and removed with vsearch. Dereplicated sequences were clustered into OTUs using SWARM, with *d* = 1 (Mahé et al., 2014). Representative sequences were then taxonomically assigned via the RDP classifier against the SILVA database (Wang et al., 2007; Quast et al., 2013). Top abundant OTUs were blasted against the NCBI 16S ribosomal RNA database. The OTUs and their closely related neighbors were aligned and screened using the V4 hypervariable region in Mothur. A maximum likelihood tree was generated in MEGA7 with 1000 bootstraps (Kumar et al., 2016).

Quantification of both SSU rRNA genes and dinitrogenase (*nifH*) genes was performed using quantitative PCR on a StepOnePlus real-time PCR platform (Applied Biosystems, CA, United States) using established methods (Gaby and Buckley, 2017; Kolton et al., 2019; Shin et al., 2019). The qPCR assay employed a PowerUp SYBR Green Master Mix (Applied Biosystems, CA, United States). Quantification of SSU rRNA was performed using the primer set 331F (5′-CCTACGGGAGGCAGCAGT-3′)/518R(5′-ATTACCGCGGCTGCTG-3′) (Muyzer and Waal, 1993). Standard curves were obtained by serial dilution of standard plasmids containing target Escherichia coli k12 SSU rRNA as the insert (2.76 × 10^3^ to 2.76 × 10^8^ copies). The running conditions were: 2 min at 50°C, 2 min at 95°C, followed by 40 cycles of 95°C for 15s, 55°C for 15s and 72°C for 1 min. The primer set used was PolF(5′-TGCGAYCCSAARGCBGACTC-3′)/PolR(5′-ATSGCCATCATYTCRCCGGA-3′) (Poly et al., 2001). Standard curves were obtained by serial dilution of standard plasmids containing target *Azotobacter vinelandii nifH* gene fragments as the insert (3.2 × 10^2^ to 3.2 × 10^7^ copies). The running conditions were: 2 min at 50°C, 2 min at 95°C, followed by 45 cycles of 95°C for 15s, 63°C for 1 min. In all experiments, negative controls containing no template DNA were subjected to the same qPCR procedure to exclude or detect any possible DNA contamination.

## Results

### Impact of Dispersant and Mixing Conditions on Hydrocarbon Biodegradation

The current experiments were conducted with freshly collected seawater, and the active microbial community immediately responded to oil addition, with no observed lag phase in activity. The oil used in the experiment was weathered with a 20% weight loss, which is considered representative of surfaced oil which is treated with dispersant *in situ* during emergency response efforts (National Research Council, 2005).

Microbial respiration was quantified as oxygen consumption and CO_2_ production (Supplementary Figure S2). In the high mixing experiment, no significant differences in respiration rate were observed between treatments, with measured oxygen consumption rates of 4.39 ± 0.64 and 4.46 ± 0.75 μmol O_2_/L/day measured in oil only and oil + dispersant treatments, respectively. Similar to the oxygen consumption results, no difference was observed in CO_2_ production rates between treatments (0.34 ± 0.01 and 0.35 ± 0.02 μmol CO_2_/L/day for oil only and oil + dispersant treatments, respectively). In the low mixing experiment, a slight increase in respiration rates was found in the dispersant treatment, but the difference was not statistically significant (2.71 ± 0.52 and 3.44 ± 0.52 μmol O_2_/L/day for oil only and oil + dispersant treatments, respectively). Rate of CO_2_ production slightly increased from 0.41 ± 0.02 to 0.49 ± 0.03 μmol CO_2_/L/day (*p* < 0.05) when dispersant was applied. Further, major nutrients, inorganic nitrogen and phosphorus, remained relatively constant during the incubations, except for nitrate/nitrite slightly increased during the last stages (after 22 days) (Supplementary Figure S3).

**FIGURE 1:**
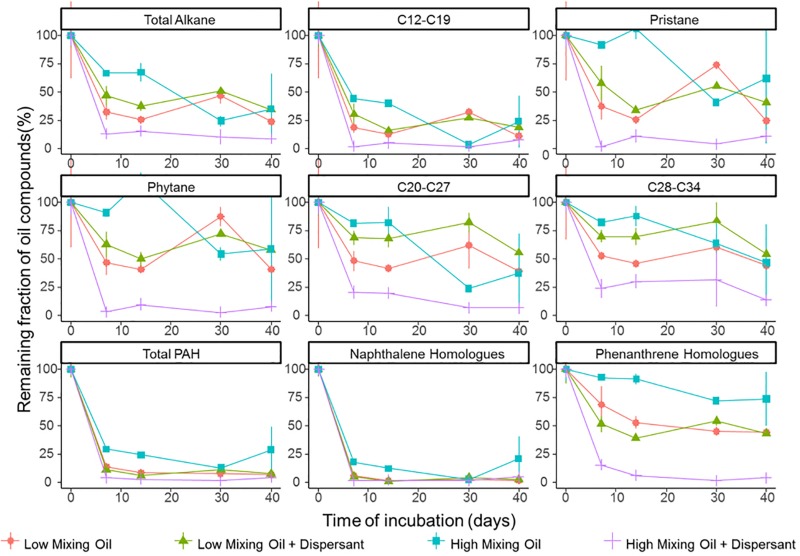
Degradation of petroleum hydrocarbons in microcosms of surface seawater. Hydrocarbon compounds are clustered based on their chemical properties and structure. Values shown are averages of duplicate measurements. Error bars are standard deviations.

Degradation significantly differed between different classes of hydrocarbon compounds (Figure 1). Low molecular weight compounds were degraded first followed by more recalcitrant compounds such as certain PAHs. First order degradation coefficients, obtained by linear regression of log transformed concentration data, suggested a significant difference between the two treatments at high mixing conditions (*p* < 0.05), while no significant difference between treatments under low mixing conditions. In all incubations, alkanes between C12 and C19 were degraded rapidly. Naphthalene and its homologs were rapidly degraded or volatilized within the first week of incubation in all treatments, whereas phenanthrene and its homologs showed a high recalcitrance to biodegradation in all incubations other than the high mixing dispersant treatment. Duplicates of the same treatment showed minor variations at the majority time points, and thus the results were representative for the scenario.

The efficacy of dispersant treatment strongly depended on the mixing condition (Figure 1). At low mixing conditions with booms, no statistical difference was observed between treatments with or without dispersant, while the degradation rate was significantly elevated in the dispersant treatment in the high mixing scenario (without booms). Under high mixing conditions, half-lives of total petroleum hydrocarbons (TPH) were 15.4 and 8.8 days for the oil only and oil + dispersant treatments, respectively (Supplementary Figure S4). The effect of dispersant on biodegradation was more pronounced for lower solubility compounds, including phenanthrene and its homologs and long chain alkanes (C ≥ 20).

Diagnostic isomer ratios were used for confirmation of both alkane and PAH degradation. The ratio for *n*-C17/pristane and *n*-C18/phytane decreased with time in all treatments (Supplementary Table S1), clearly indicating the loss of alkane compounds was due to degradation (De Jonge et al., 1997). For PAH degradation, preferential degradation of 2-methylphenanthrene over 1-methylphenanthrene was previously established as an indicator of biodegradation (Bastiaens et al., 2000; Leys et al., 2005; Lamberts et al., 2008). The ratio of 2-methylphenanthrene to 1-methylphenanthrene increased with time in the high mixing condition treatments (Supplementary Figure S5) providing further evidence of biodegradation (58).

### Response of the Total and Active Microbial Communities

**FIGURE 2:**
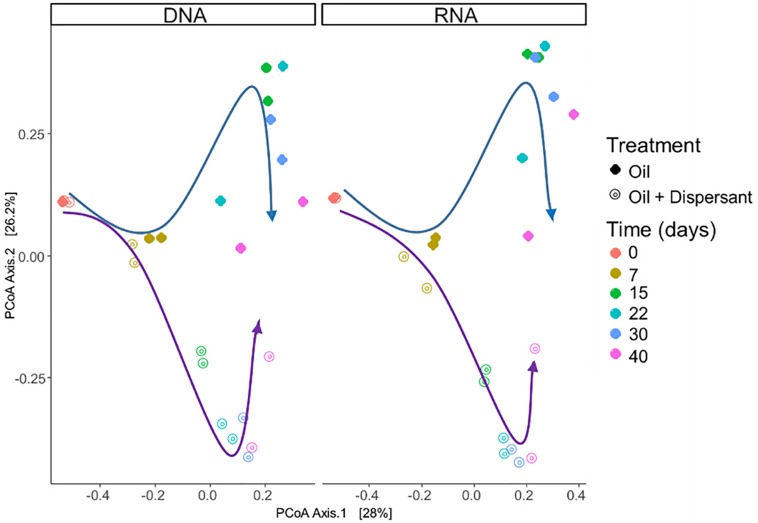
Comparison of microbial community composition with incubation time and treatment in seawater microcosms for both high mixing treatments. **Left** and **right** panels show microbial community composition from sequencing of SSU rRNA genes in DNA and RNA extracts, respectively. Arrows highlight the changes in microbial community composition: blue arrows indicate oil treatments, purple arrows indicate the oil + dispersant treatments. Similarities between microbial communities are displayed as the Bray–Curtis distance metric on a PCoA plot.

**FIGURE 3:**
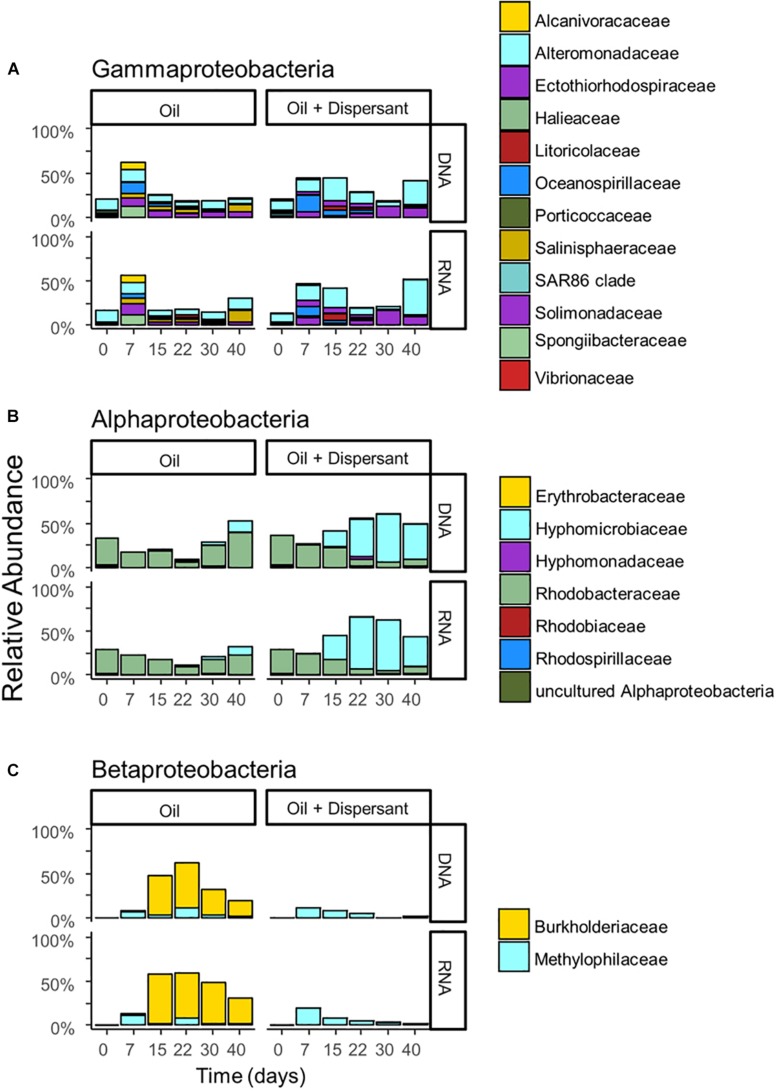
The relative abundance of **(A)** *Gammaproteobacteria*, **(B)** *Alphaproteobacteria*, and **(C)** *Betaproteobacteria* with incubation time and treatment for high mixing treatments. Barplots show mean value of duplicated samples. Taxa are grouped at the family level and relative abundance is calculated relative to total sequences.

Microbial communities were characterized in high mixing condition treatments to mimic the dynamics of microbial populations that carry out biodegradation of completely dispersed oil in seawater. We used RNA sequences as a proxy to characterize the metabolically active microbial populations in oil-contaminated marine ecosystems. Similar patterns in community compositions were observed over time at the RNA and DNA level (Figures 2, 3, and Supplementary Figure S6), indicating that the majority of groups detected are metabolically active. According to PERMANOVA analysis (Anderson, 2005), both time and treatment were statistically significant factors that shaped microbial communities (*p* < 0.05).

**FIGURE 4:**
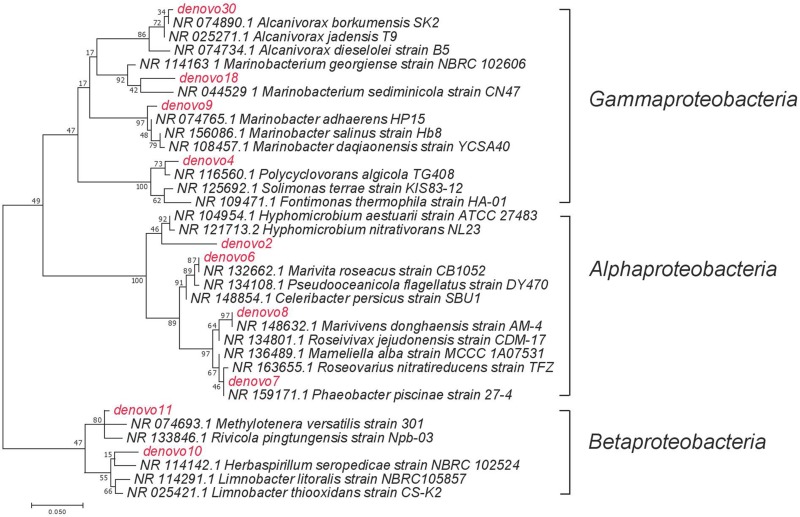
The maximum likelihood phylogenetic tree of selected dominant OTUs. Names in red indicated the dominant OTUs from the current study, names in black indicated the closely related taxa that has been previously reported.

A succession of microbial populations, dominated by those affiliated with known hydrocarbon-degrading bacterial groups, was observed in parallel with the chemical evolution of petroleum hydrocarbon compounds. Microbial community composition was consistent between duplicates of the same treatment (Figure 2). Community composition of the oil only and oil + dispersant treatments clearly diverged substantially by day 15. Bray–Curtis distance metrics showed that the two treatments diverged until 2–3 weeks of incubation and then converged by the end of the 40 day incubation, indicating a more similar composition of both total and metabolic active community by the end of the experiment. A large fraction of the retrieved sequences could not be assigned at the genus level, indicating that most microbial populations that develop in dispersed oil remain uncharacterized. Microbial communities during the first 2 weeks in all treatments were dominated by the class *Gammaproteobacteria* (Figure 3), which contributed up to 63% (DNA) and 58% (RNA) relative sequence abundance in oil only treatments, and 44% (DNA) and 45% (RNA) in dispersant treatments. Succession over time in individual microbial populations followed the changes in hydrocarbon chemistry. Typical oil degraders observed during oil inputs, such as *Alcanivorax* (denovo30) were observed within in the first week of incubation and then disappeared in the community (Figure 4). A dominant group (denovo4) that was closely related to the *Polycyclovorans algicola* of the family *Solimonadaceae* (Figure 3A), showed high relative abundance during the first 2 weeks of the incubation, contributing up to 22.3% in individual samples. A bloom of the family *Oceanospirilliceae* was also observed on day 7 in both treatments. The dominant OTU (denovo18) showed high sequence identity to *Marinobacterium marisflavi*. The group *Alteromonadaceae* was also abundant in both cases, and the closest match of the dominant OTU (denovo9) was closely related to the *Marinobacter adhaerens*.

An OTU (denovo1) within the *Burkholderiaceae* of the *Betaproteobacteria* dominated in oil only treatments during the middle to late stages of incubation, comprising up to 51% of the total DNA library on day 22 and 56% of the total RNA library on day 15 (Figure 3C). Another family of the *Betaproteobacteria* group responding to the treatment was the *Methylophilaceae*. The dominant OTU (denovo11) of the family was closely related to the *Rivicola pingtungensis* and *Methylotenera versatilis*.

Two major families, *Hyphomicrobiaceae* and *Rhodobacteraceae*, of the *Alphaproteobacteria* were showed high relative abundance within the microbial community (Figure 3B). Multiple OTUs, represented by denovo8, related to the *Rhodobacteraceae*, were abundant in the early stages of the incubation, contributing up to 22 and 24% at the RNA level in both oil and dispersant treatments at day 7, respectively. Another set of OTUs, including denovo6, within the *Rhodobacteraceae* dominated the communities during the latter stages of incubation in oil only treatments. An OTU within the family *Hyphomicrobiaceae* (denovo2) dominated the microbial community from day 15 in the oil + dispersant treatment, constituting up to 55 and 57% at the DNA and RNA level, respectively (Figure 3B). A bloom of *Hyphomicrobiaceae* was observed at day 15, when both naphthalene and phenanthrene homologs were depleted in the oil + dispersant treatment. Meanwhile, an increase in relative abundance of the family *Hyphmicrobiaceae* emerged in the oil only treatment on day 40.

**FIGURE 5:**
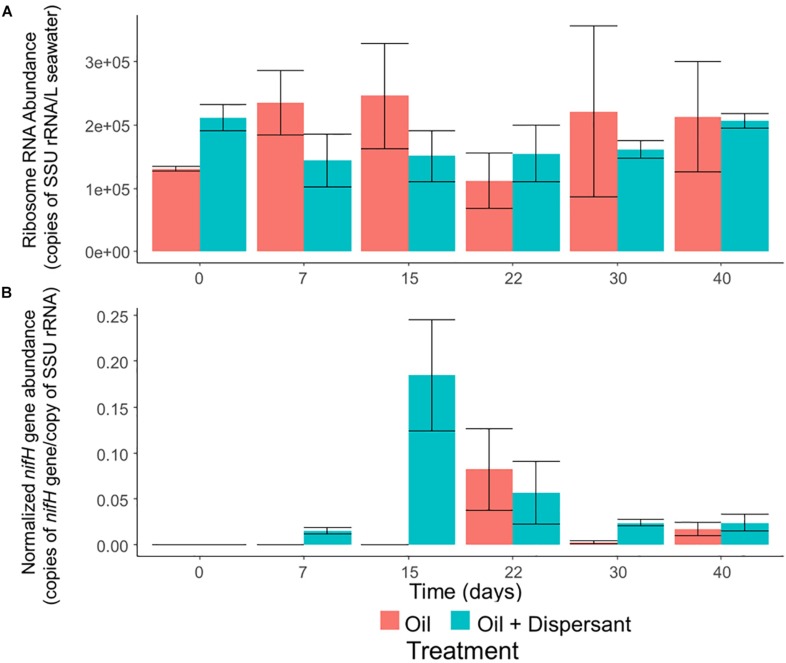
The abundance of overall bacteria and nitrogen-fixing prokaryotes in high mixing seawater microcosms as determined by quantitative PCR of **(A)** SSU rRNA genes and **(B)** *nifH* genes normalized to SSU rRNA genes. Error bars represent the standard deviation of duplicates.

Microbial abundance, as determined by qPCR of SSU rRNA genes, was constant throughout all incubations (Figure 5A). In contrast, our qPCR results suggest that certain ecosystem functions are impacted by oil and dispersant treatment. The relative abundance of nitrogenase gene (*nifH*), which is a widely used molecular marker of nitrogen fixation (Gaby and Buckley, 2014), was up-regulated during the middle stages of incubation (Figure 5B), and the maximum abundance of *nifH* in the oil + dispersant treatments was over twice that in the oil only treatments. The abundance of *nifH* in the oil + dispersant treatments peaked on day 15, comprising 18% of the community as estimated by normalizing to the abundance of SSU rRNA genes. In the oil only treatments, a maximum abundance of 8% was observed at day 22.

## Discussion

The primary objectives of this study were to investigate the influence of mixing condition on the ability of dispersant to enhance PHC biodegradation as well as to identify dispersant impacts on metabolically active microbial communities in surface seawater from the Gulf of Mexico. Boom treatments were used to simulate conditions when an oil slick is not treated with dispersant or under conditions when there is inadequate turbulence to mix dispersant with oil in the slick. The simulated oil slick remained in booms and was visible throughout the 40-day incubation with and without dispersant application. Treatments without booms simulated a scenario, where the dispersant was efficiently mixed with oil by turbulence and thus biodegradation was further enhanced. Mixing energy in the surface ocean is defined by dissipative power, which ranges from 0 W/kg without wind or wave action to 1 W/kg during catastrophic storms such as a hurricane or typhoon (Deane et al., 2016). Our low mixing treatment should resemble the windless/waveless condition, whereas the high mixing treatment mimics the top few meters of surface ocean exposed to substantial wind or wave mixing. Quantification of turbulence energy is beyond the scope of this investigation and warrants further study.

### Impact of Dispersant Application on the Rates and Controls of Hydrocarbon Biodegradation

In the current study, addition of low concentrations of oil that mimic response conditions did not stimulate microbial respiration in the mesocosms. This result was corroborated by previous work, which showed no or minor changes in oxygen consumption upon oil + dispersant treatment (McFarlin et al., 2014), and respiration rates from our study are comparable to those measured in the surface oil slick generated by the Deepwater Horizon (DWH) discharge (ranging from 4.8 to 16.3 μmol O_2_/L/day) (Edwards et al., 2011). Although the O_2_ consumption to CO_2_ production ratio was relatively high (∼13), this could be explained by the formation of oxygenated PHCs and other intermediates. Biodegradation was shown to introduce oxygen into PHCs and form polarized intermediates, which are not able to be resolved by GC measurement (Aeppli et al., 2012). Since dispersant plus mixing led to enhanced biodegradation, these results indicate that oil biodegradation represented only a portion of the indigenous respiration occurring in our incubations. Respiration rates observed in the current study are corroborated by previous work, which suggested an average background respiration of 2.2 μmol O_2_/L/day in surface seawater in the GOM (Edwards et al., 2011). Similarly, the minimal changes in bacterial abundance and major nutrients observed in the current study also agreed with previous experiments conducted at low concentrations of oil expected during emergency response efforts (Edwards et al., 2011; McFarlin et al., 2014; Brakstad et al., 2015). Elevation in nitrate/nitrite concentrations, observed during the later stages of incubation, can be explained by the release of mineralized NH_4_^+^ followed by nitrification to NO_3_^–^ when labile carbon substrate availability decreased. Overall, our observations indicate that overall respiration is neither inhibited nor enhanced by dispersant, and dispersant does not appear to stimulate the total growth of microbial communities at concentrations that resemble those expected during *in situ* response efforts. These findings agree with field observations during the DWH spill, where bacterial biomass remained constant within and outside the weathered surface oil slick (Edwards et al., 2011). In contrast, at similar oil concentrations, Wang et al. (2016) reported a fivefold increase in microbial abundance during incubation of deeper waters (collected from 1500 m water depth) amended with dispersed oil. Wang et al. (2016) may have observed growth due to the fact that their seawater mesocosms were amended with fresh crude oil along with dispersant, whereas our study employed weathered Macondo surrogate oil, which is more recalcitrant to biodegradation, as it contains a decreased portion of low molecular weight (LMW) compounds. Depletion of the labile portion of oil will reduce substrate quality and therefore diminish bacterial growth efficiency (Eiler et al., 2003). Alternatively, nutrient concentrations were an order of magnitude higher in the deep seawater used by Wang et al. (2016) which could have supported enhanced growth.

In this study, biodegradation followed previously reported patterns, in that labile compounds were degraded first followed by more recalcitrant compounds, especially for multi-methylated PAHs (Hu et al., 2017). The loss of PHCs in low mixing treatments appeared to be faster than the high mixing oil only treatment. Since experimental conditions were consistent between two oil only treatments, this could be explained by the fact that surface water was collected at a different time for each treatment and thus the results could reflect the presence of a different microbial populations, which warrants further study.

The differences in biodegradation rates among the treatments suggested that when directly applied to the oil slick without sufficient mixing, dispersant may not enhance hydrocarbon degradation. Under high mixing conditions, the application of dispersant enhanced the degradation of nearly all hydrocarbon groups. As a result, the decreased half-life of the total PHCs was comparable with previous measurements of 14 days for chemically dispersed oil in surface water (Prince and Butler, 2014) as well as degradation rates measured in simulated oil plumes (Hazen et al., 2010a; Prince et al., 2013; Wang et al., 2016). In addition, our results are in agreement with model predicted half-lives (Prosser et al., 2016). The effectiveness of the dispersant was more pronounced on recalcitrant compounds, possibly due to their low solubility, as these compounds were likely to remain with droplets. Thus, dispersant application could enhance degradation by decreasing droplet size and increasing the surface-to-volume ratio of the oil-water interface (Brakstad et al., 2014, 2017; Prince et al., 2017).

A subset of previous work has observed no increase in hydrocarbon degradation after dispersant application (Lindstrom and Braddock, 2002; Macías-Zamora et al., 2014) or concluded that microorganisms use dispersant as a carbon substrate and therefore attenuate hydrocarbon degradation (Lindstrom and Braddock, 2002; Kleindienst et al., 2015b). Inconsistencies in the effects of dispersant on hydrocarbon degradation should be interpreted with caution and may be attributed to methodological considerations. Studies have often employed oil, nutrient amendments, or dispersant-to-oil ratios that are far from *in situ* conditions expected during response efforts after a spill (Lee et al., 2013). Experiments that employ a water accommodated fraction (WAF) or a chemically enhanced water accommodated fraction (CEWAF) are difficult to compare to those in which oil and dispersant are directly added to seawater. The WAF method often excludes floating oil, which is likely to have a major impact on the activity of microbial communities in the incubations (Prince et al., 2016a). For example, using WAF and CEWAF treatments in seawater incubations, previous studies suggested that dispersant could suppress hydrocarbon degradation by selecting for dispersant degrading bacteria and against the most effective hydrocarbon degraders (Kleindienst et al., 2015b, 2016). However, it is unclear what portion of the amended oil was contained in the incubations and it is possible that oil droplets that were not entrained during the production of WAF or CEWAF would be excluded and therefore alter the availability of oil for biodegradation (Prince et al., 2016a). A higher oil carbon content in the WAF treatment could explain why microbial activity was higher relative to that of the CEWAF treatment in the study (Kleindienst et al., 2015b).

When dispersant is applied to oil slicks at the ocean surface, undispersed oil will remain in the slick, potentially hindering biodegradation. Therefore, this study focused on the comparison of a simulated oil slick to fully dispersed oil (Bejarano et al., 2013). The results confirm that dispersant application requires sufficient mixing power to disperse oil slicks (National Research Council, 2005; Prince, 2015). With sufficient mixing, dispersant enhances hydrocarbon degradation. At near *in situ* oil concentrations, the dispersant enhances hydrocarbon degradation under sufficient mixing condition. In contrast, dispersant application did not lead to a significant increase in oil degradation under the low mixing condition. Other environmental parameters, such as oxygen and nutrient availability, are unlikely to limit biodegradation at low oil concentrations (Lee et al., 2013).

### Response of the Metabolically Active Microbial Communities to Dispersant Application

Changes in microbial communities in general paralleled PHC degradation patterns. The correspondence between total and active microbial community compositions suggested rapid turnover of microbial biomass. Shifts in community beta diversity with time suggested recovery from oil perturbation at the end of the experiment, agreeing with previous literature (Brakstad et al., 2017). The dominance of *Proteobacteria* was expected, as this group was often observed in previous PHC contaminated sites (Hazen et al., 2010b; Kostka et al., 2011; Valentine et al., 2014). The *Gammaproteobacteria* group contains many known aerobic hydrocarbon-degrading bacteria, such as *Alcanivorax*, that were shown to rapidly respond to oil deposition in marine ecosystems (Harayama et al., 1999; Kasai et al., 2002; Gertler et al., 2009; Hazen et al., 2010a; Kostka et al., 2011; Beazley et al., 2012). For example, similar to our previous field observations in Pensacola beach sands that were impacted by oil from the DWH disaster, a bloom of *Alcanivorax dieselolei* was observed in the oil treatments of this study (Figure 3A; Kostka et al., 2011). The *Alcanivorax* observed in the current study was closely related to the *Alcanivorax borkumensis*, a type strain widely studied for hydrocarbon biodegradation (Yakimov et al., 1998; Naether et al., 2013). However, *Alcanivorax* was not observed in abundance in the dispersant treatments, which may indicate that Corexit 9500 inhibits the adherence and growth of *Alcanivorax*, as suggested in a previous study (Bookstaver et al., 2015). Alternatively, dispersant application may have dramatically enhanced alkane availability and resulted in such a rapid response of *Alcanivorax* that by our first sampling point, day 7, this group was already succeeded by other taxa.

The genus *P. algicola* was suggested to utilize a wide range of both aliphatic and PAH compounds (Gutierrez et al., 2013, 2015). The substrate range of this group would account for its presence throughout the incubations, including when labile alkanes and low molecular weight PAHs were depleted. The presence of this OTU (denovo4) in both treatments suggested it is insensitive to dispersant. The response of the *Oceanospirillaceae* and *Alteromonadaceae* shown here agreed with observations of *in situ* microbial communities in contaminated seawater during the DWH disaster. Both groups contain known hydrocarbon-degrading bacteria that increased in relative abundance in response to oil spills in various environments (Deppe et al., 2005; Atlas et al., 2015; Yang et al., 2016; Techtmann et al., 2017).

Degradation occurred at a slower rate in the oil only treatment, in concurrence with a more prolonged bloom of microbial populations capable of degrading low molecular weight PAHs, such as members of the *Burkholderiaceae*. The dominance of this group is noteworthy, as members of the *Betaproteobacteria* usually comprise a relatively low percentage of marine bacterioplankton. The most abundant OTU, denovo1, was closely related (97% similarity) to *Limnobacter litoralis* KP1-19 (Lu et al., 2011). Previous work detected the essential genes for aromatic compound degradation in *Limnobacter* (Vedler et al., 2013), and this microbial group responded to crude oil addition in concurrence with the presence of PAHs (Canul-Chan et al., 2017). The absence of this group in the dispersant treatment may suggest its growth was inhibited by the presence of dispersed oil or an inability to compete with other PAH degraders in dispersed oil. The *Methylophilaceae* also increased in relative abundance *in situ* during the DWH discharge (Kessler et al., 2011; Eyice et al., 2015). This group contains well known methylotrophic bacteria, and some members are putative hydrocarbon-degraders (Thompson et al., 2017). Alternatively, Joye et al. (2014) suggested that these C1-oxidizers could be responding to the elevated DOM generated by the hydrocarbon-degraders.

The presence of the *Rhodobacteraceae* was constant throughout the incubation. The dominant populations, however, shifted with incubation time, and possibly with substrate availability. At early stages of incubation, multiple OTUs were enriched within the community, which could be responsible for the degradation of low molecular weight PAHs (Gutierrez et al., 2011). The closely related strain to dominant OTU denovo8 was *Marivivens* sp. JLT3646, which was suggested to possess the potential to degrade monoaromatic compounds based on genome annotation (Chen et al., 2018).

After depletion of all low molecular weight PHCs, other members within the *Rhodobacteraceae* were enriched during the latter stages of incubation, in agreement with previous observations attributing the degradation of more recalcitrant hydrocarbons to members of the *Rhodobacteraceae* (Kostka et al., 2011). The dominant OTU, denovo6, showed high sequence similarity to *Marivita roseacus* (99%) of the *Roseobacter* lineage (Budinoff et al., 2011). This lineage is a ubiquitous marine bacterial group that associates with phytoplankton cells or organic particles in the surface ocean (Slightom and Buchan, 2009). This cell-surface interaction may explain the abundance of this lineage in the oil treatment, possibly attaching to the oil slick, while the absence of slick in the dispersant treatment would be less favorable. Some phytoplankton-associated heterotrophic bacteria utilize hydrocarbons, both alkanes and PAHs, as they grow on natural hydrocarbons produced or accumulated by phototrophs (Gutierrez et al., 2011; Lea-Smith et al., 2015). One recent study suggests that members of the *Roseobacter* group have the potential to degrade aromatic hydrocarbons (Rodrigo-Torres et al., 2017). In fact, Klotz et al. isolated a new *Roseobacter* strain, *Tritonibacter horizontis*, from oil contaminated surface water collected in the Gulf of Mexico during the DWH discharge (Klotz et al., 2018). The strain demonstrated the ability to utilize hydroxylated and substituted aromatic compounds but cannot grow on alkanes (Giebel et al., 2016). Thompson et al. (2017) also found that members of this lineage were enriched by oil, especially in the presence of phytoplankton.

No strains matching sequences in the NCBI library fell within the same genus with denovo2. The closest neighbor was *Hyphomicrobium vulgare*, with only 93% sequence similarity, which suggested they belong to the same family, *Hyphomicrobiaceae*. Members of the *Hyphomicrobiaceae* were previously demonstrated to degrade hydrocarbons in pure culture and were enriched in previous studies of oil contaminated sites (Ozaki et al., 2006; Lafortune et al., 2009; Fahrenfeld et al., 2014). For example, Rodrigues et al. (2017) found members from this group were associated with both fluoranthene and pyrene addition, indicating their ability to degrade more recalcitrant high molecular weight PAHs. In the current study, the dominance of the *Hyphomicrobiaceae* was observed at different time points between oil only and oil + dispersant treatments. This phenomenon suggested a similar community response to reduced availability of low molecular weight hydrocarbons affected by different treatments.

Results of the current study indicate that dispersant application did not substantially alter bacterial abundance during PHC degradation, which supports previous observations in the surface oil slick after the DWH spill (Edwards et al., 2011). Meanwhile, enrichment in *nifH* genes suggested nutrient limitation during incubation. Since oil is depleted in major nutrient elements (i.e., nitrogen and phosphorus), biodegradation of this carbon-rich substrate has long been associated with nutrient limitation in marine environments (Atlas, 1981). For example in a metagenomic time series, an increase in the abundance of genes encoding nitrogen fixation and other nutrient acquisition pathways correlated with the abundance of genes for hydrocarbon degradation in oil-contaminated beach sands impacted by the DWH spill (Rodriguez-R et al., 2015). The nitrogen fixation process was followed by the nitrification mentioned earlier. Neither nitrification nor nitrogen fixation processes were shown to be associated with specific OTUs. Our results provide quantitative evidence that links nitrogen fixation to hydrocarbon degradation in microbial communities of surficial seawater, suggesting that these planktonic communities undergo nutrient limitation in response to oil contamination in coastal ecosystems (Rodriguez-R et al., 2015).

## Conclusion

The results of this study indicate that when applying dispersant to an oil slick, biodegradation may not be substantially enhanced unless sufficient mixing is provided. When the simulated slick was sufficiently dispersed, a higher rate of removal was observed for more recalcitrant hydrocarbon compounds (such as methylated phenanthrene), suggesting that surface area available for microbial colonization is a primary factor limiting hydrocarbon degradation, and the application of dispersant will likely alleviate this constraint. While microbial growth and respiration were not substantially altered between treatments, RNA analysis revealed that dispersant application resulted in pronounced changes to the composition of metabolically active microbial communities. The quantitative increase in nitrogen-fixing members of the microbial community suggests a selection pressure for nitrogen fixation, likely indicating the robust response of the indigenous microbial communities to a readily biodegradable nitrogen-poor substrate. In order to improve model predictions and the bioremediation of dispersed oil during emergency response efforts, future study is warranted on the coupling of biodegradation to nitrogen fixation.

## Data Availability Statement

The datasets generated for this study can be accessed from NCBI, PRJNA434233.

## Author Contributions

XS: conceptualization, methodology, formal analysis, investigation, writing – original draft, visualization, and validation. LC and EM: methodology and investigation. IR: methodology, investigation, and resources. DH: resources and funding acquisition. JK: conceptualization, resources, writing – review and editing, supervision, and funding acquisition.

## Conflict of Interest

The authors declare that the research was conducted in the absence of any commercial or financial relationships that could be construed as a potential conflict of interest.
