# Supplementary material for: Dispersant Enhances Hydrocarbon Degradation and Alters the Structure of Metabolically Active Microbial Communities in Shallow Seawater From the Northeastern Gulf of Mexico
Source: Front Microbiol. 2019 Oct 18;10:2387. doi: 10.3389/fmicb.2019.02387 (PMC6842959; doi:10.3389/fmicb.2019.02387)
Supplement: Supplementary file 1 [file Data_Sheet_1.docx]

SUPPORTING INFORMATION

**Dispersant enhances hydrocarbon degradation and alters the structure of metabolically active microbial communities in shallow seawater from the northeastern Gulf of Mexico**

Xiaoxu Sun,^1,2^ Lena Chu,^3^ Elisa Mercando,^3^ Isabel Romero,^4^ David Hollander,^4^ and Joel E Kostka^1,3,^ *

^1^ School of Earth and Atmospheric Sciences, Georgia Institute of Technology, Atlanta, GA, United States

^2^ Guangdong Key Laboratory of Agricultural Environment Pollution Integrated Control, Guangdong Institute of Eco-Environmental Science & Technology, Guangzhou, China

^3^ School of Biological Sciences, Georgia Institute of Technology, Atlanta, GA 30332, United States

^4^ ﻿ College of Marine Science, University of South Florida, Saint Petersburg, FL, United States

* Corresponding author

Joel E Kostka,

Room 225, Cherry Emerson Bldg.

310 Ferst Drive

Atlanta, Georgia, 30332-0230

Email: [joel.kostka@biology.gatech.edu](mailto:joel.kostka@biology.gatech.edu)

7 Pages

5 Figures

1 Table


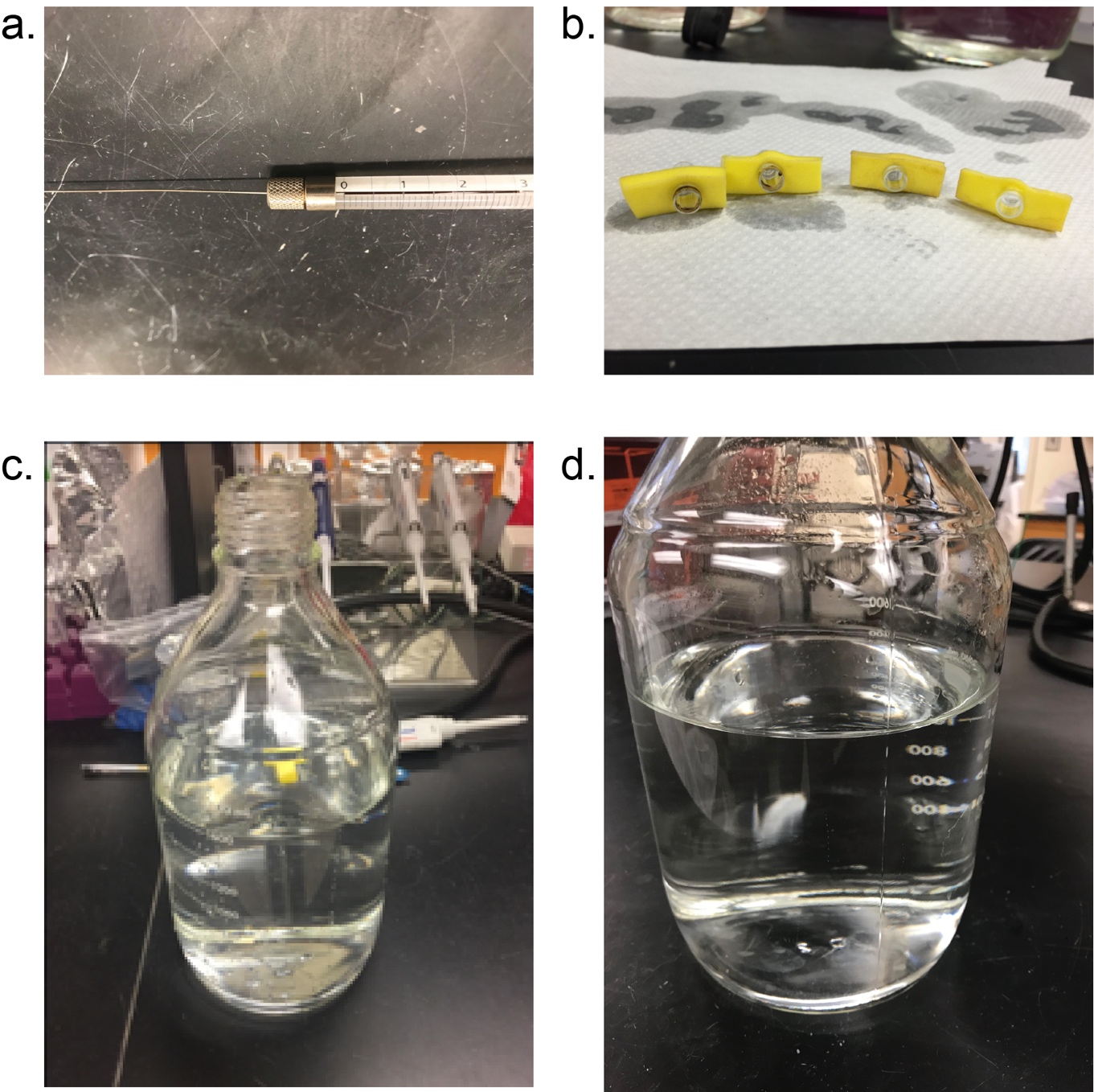


Figure S1. Images illustrating the experimental setup: a. Analytical needle for oil/ dispersant addition. b. Booms used to constrain the oil as a surface slick. c. Experiment setup for both treatments under the low mixing condition and the oil only treatment under the high mixing condition. d. Dispersant treatment that simulated complete dispersion of oil and dispersant under high mixing condition.


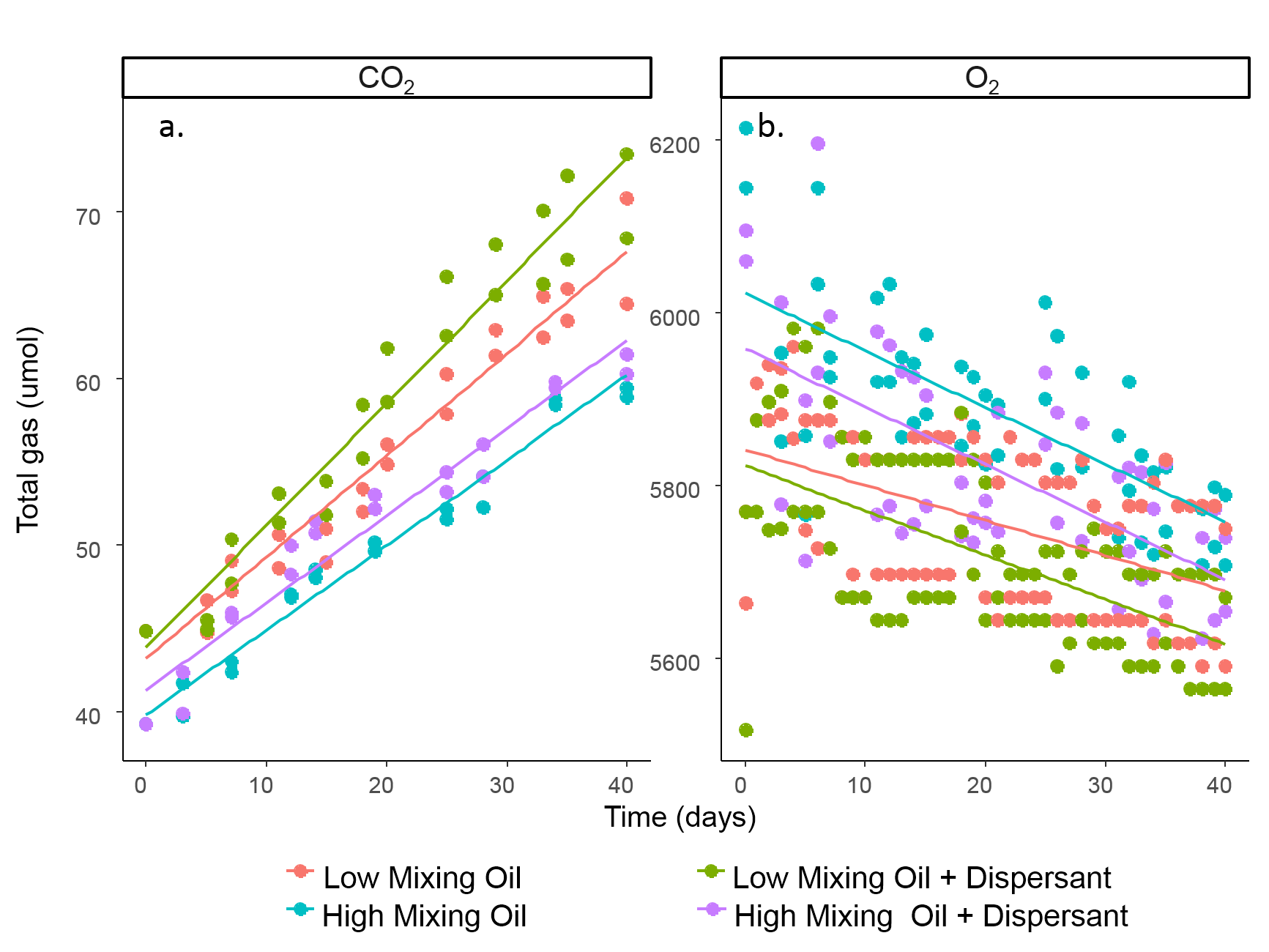
Figure S2. Microbial respiration as determined by carbon dioxide accumulation and oxygen consumption in seawater microcosms. The left and right panels show total carbon dioxide and total oxygen in the microcosms, respectively. Lines are fitted linear regressions.


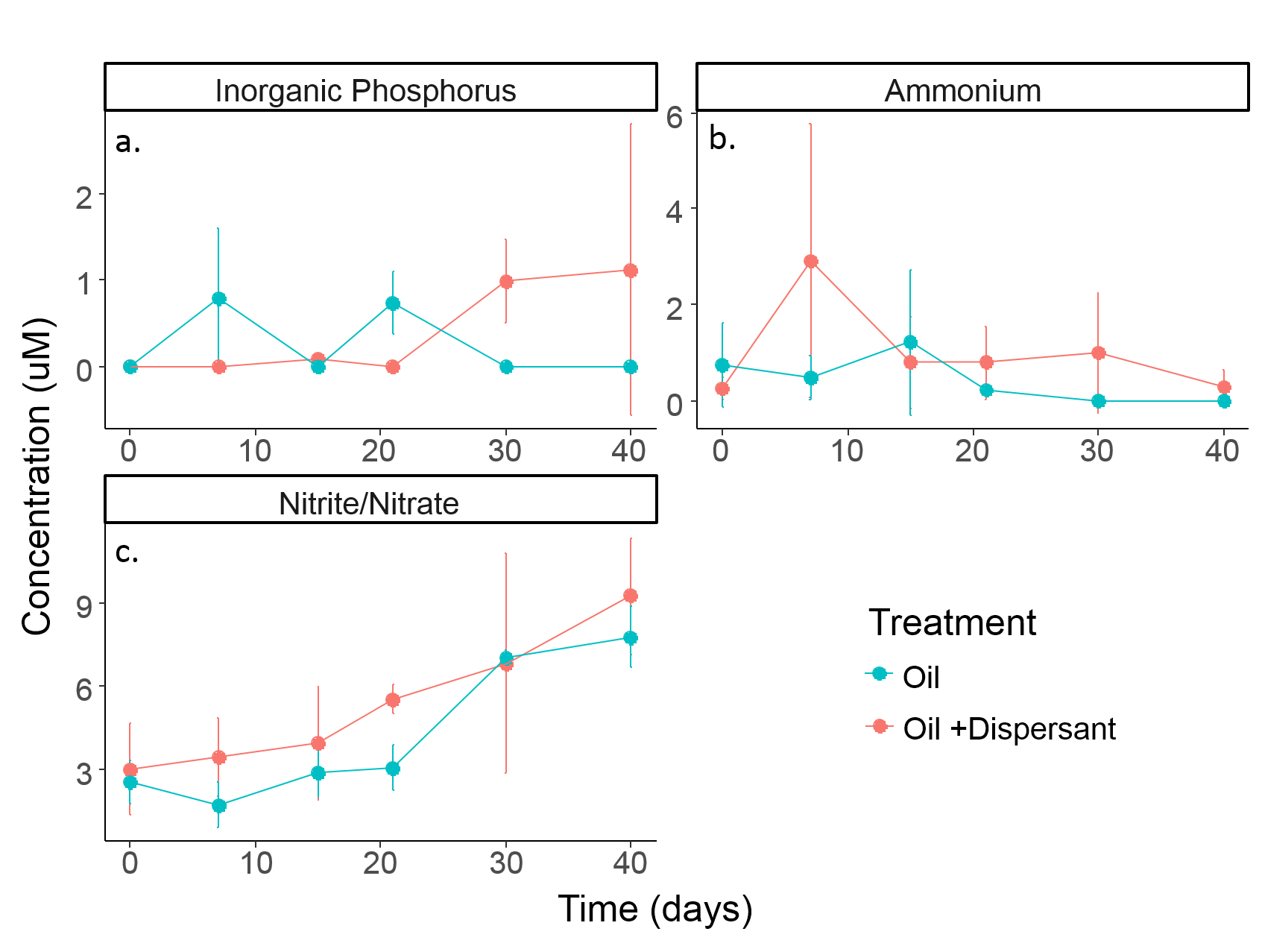


Figure S3. Concentrations of major inorganic nutrients in the high mixing treatments. Values shown are averages from four replicate measurements. Error bars are standard deviations.


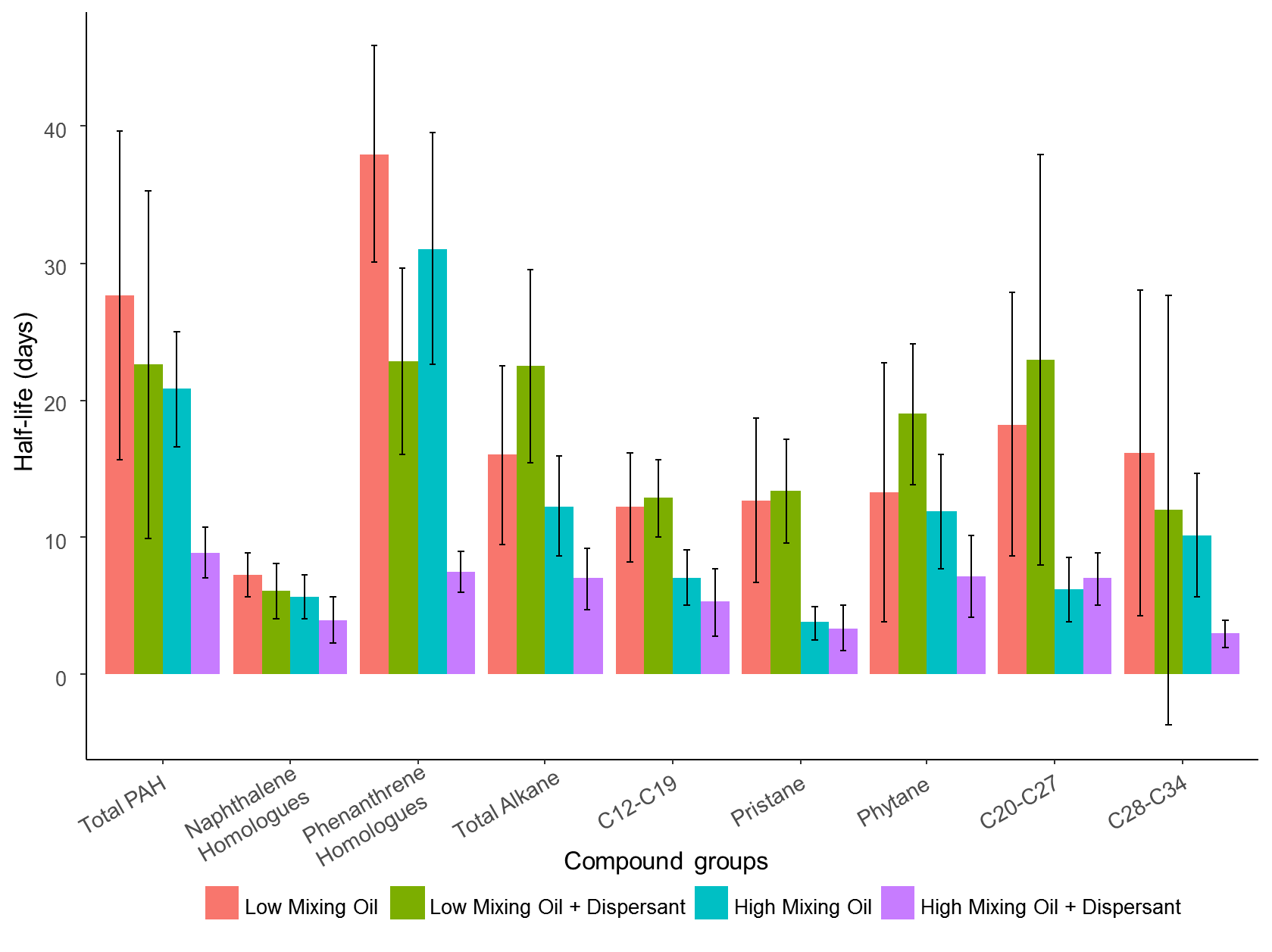


Figure S4. First order kinetic half-lives for hydrocarbon removal. The half-lives are calculated using transformed data to fit a first order kinetic equation and error bars represent the standard deviation of the fitted curve. This method is less accurate for labile compounds in the high mixing dispersant treatment due to their potential rapid removal before the first measurement.

Figure S5. 2-Methylphenanthrene/1-Methylphenanthrene ratio. Colors indicate different treatments. Increases in values suggest biodegradation in the samples.


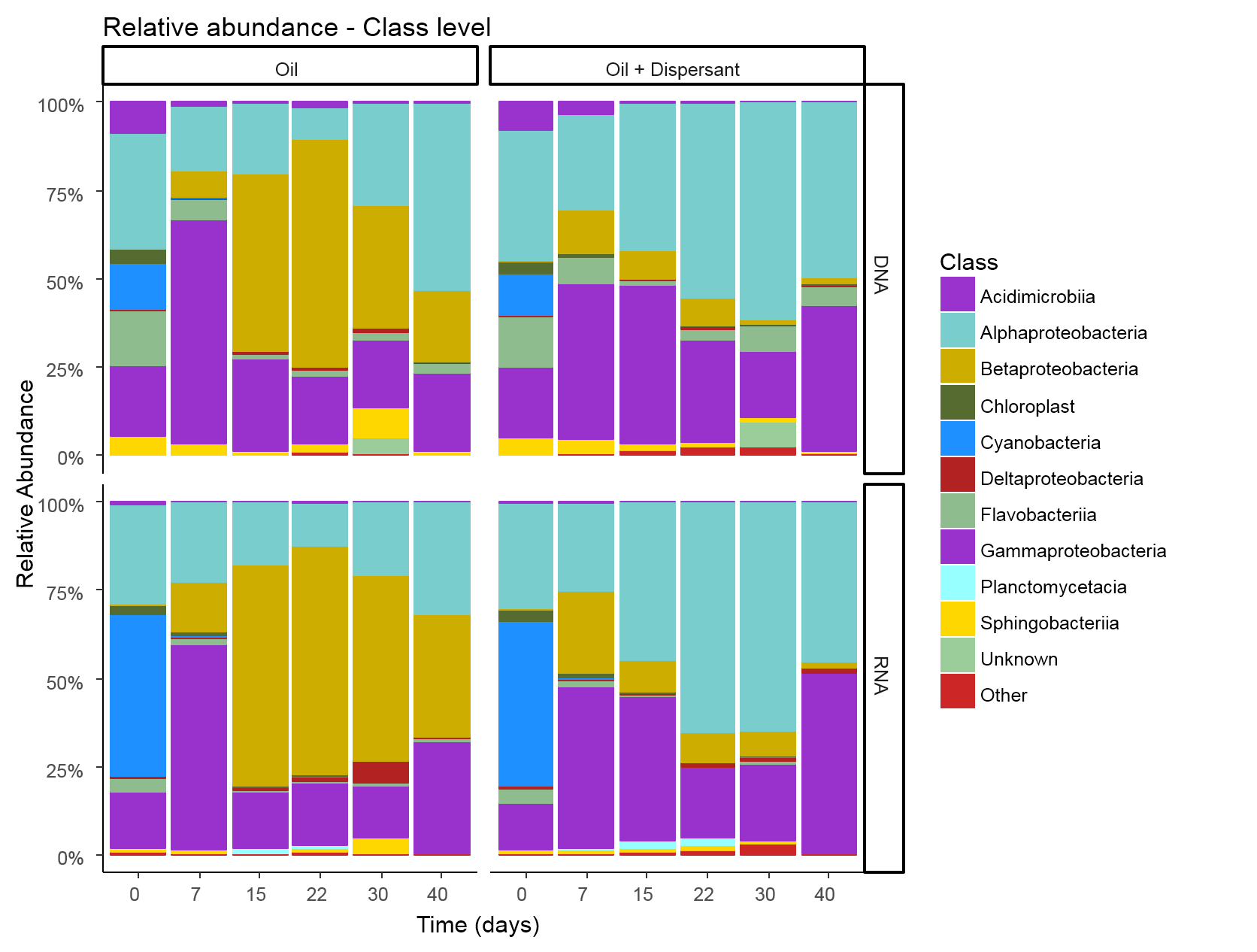


Figure S6. The relative abundance of microbial groups of high mixing treatments at the phylum to class level with incubation time and treatment. Barplots show mean values of duplicated samples. Taxa are grouped at the class level and relative abundance is calculated relative to total sequences retrieved for each class.

Table S1 C17/pristane and C18/phytane ratios determined from HC analysis of microcosm samples by GC-MS

| Mixing condition | Treatment | C17/pristane | C18/phytane |
| --- | --- | --- | --- |
| Low-Mixing | T0 | 1.8 ± 0 | 2.4 ± 0.1 |
|  | Oil | 1.4 ± 0 | 1.8 ± 0 |
|  | Oil + Dispersant | 1.1 ± 0.5 | 1.4 ± 0.1 |
| High-Mixing | T0 | 1.4 ± 0.1 | 1.8 ± 0.2 |
|  | Oil | 0.3 ± 0.4 | 0.6 ± 0.6 |
|  | Oil + Dispersant | 0.8 ± 0.7 | 1.5 ± 0.9 |
